# Nonalcoholic Wernicke's Encephalopathy Associated with Unintentional Weight Loss, Cholecystectomy, and Intractable Vomiting: The Role of Dual Thiamine and Corticosteroid Therapy

**DOI:** 10.1155/2014/430729

**Published:** 2014-01-19

**Authors:** Vivek Verma, Chenell Donadee, Leslie Gomez, Marina Zaretskaya

**Affiliations:** ^1^Department of Medicine, UPMC Mercy Hospital, Pittsburgh, PA 15219, USA; ^2^Division of Critical Care Medicine, UPMC Mercy Hospital, Pittsburgh, PA 15219, USA; ^3^Division of Neurology, UPMC Mercy Hospital, 1350 Locust Street, Suite 402, Pittsburgh, PA 15219, USA

## Abstract

A 23-year-old male with one month of intractable vomiting, subsequent cholecystitis status post cholecystectomy, and overall 40-pound weight loss over the last few months presented with altered mental status and seizures. MRI showed signal abnormalities involving the hypothalamus, periaqueductal gray matter, 4th ventricle, and bilateral thalami, indicative of Wernicke's encephalopathy. The patient was started on empiric IV thiamine and methylprednisolone; thiamine levels were subsequently found to be low. Infectious disease workup was negative. Within a few days of this therapy, the patient's neurological status steadily improved with increased responsiveness and communication. Repeat MRI 7 days after admission showed significant resolution of the signal abnormalities. Over the next several weeks the patient became fully conversational, cognitively intact, and increasingly ambulatory. Nonalcoholic Wernicke's encephalopathy is rare; there have been reports relating it separately to vomiting and invasive surgery. In this case report, we associate it with both recurrent vomiting and minimally invasive cholecystectomy. We also discuss combinatorial therapy of thiamine and corticosteroids, which is poorly defined in the literature. Though there is no consensus-based optimal treatment of Wernicke's encephalopathy, this adds to the discussion of using dual therapy and supports that the use of empiric corticosteroids does not harm the patient.

## 1. Introduction

Wernicke's encephalopathy manifests as a result of thiamine deficiency, causing neurological damage in multiple parts of the brain and brainstem [1]. Though most cases of this condition happen in alcoholics, a nontrivial proportion does happen in nonalcoholics. Because of the strong association with alcoholics, however, Wernicke's encephalopathy is often not entertained as a possible differential diagnostic condition.

In nonalcoholic patients, Wernicke's encephalopathy is most commonly associated with cancer, hyperemesis gravidarum and vomiting, starvation/malnutrition, and AIDS [2]. Another major cause of this condition includes a spectrum of gastrointestinal surgeries, but the vast majority of cases are specifically associated with bariatric surgeries [3].

Hence, in nonalcoholic patients with neurological symptoms concerning for Wernicke's encephalopathy, risk factors for nonalcoholic Wernicke's encephalopathy must be carefully probed. In the present case, idiopathic weight loss and minimally invasive cholecystectomy along with postoperative intractable vomiting were the main risk factors for development of Wernicke's encephalopathy. To our knowledge, there have been no reports of these multiple risk factors simultaneously being associated with Wernicke's encephalopathy. The present case also discusses combinatorial therapy for this condition with corticosteroids and thiamine, something which has garnered little attention in the literature and needs to be further addressed. We highlight with this experience that though it is difficult to delineate the precise effect of corticosteroids, administering them acutely and empirically is not harmful to the patient.

## 2. Case Presentation

A 23-year-old Caucasian male with past medical history of asthma was in normal health after a tonsillectomy in May 2013, when he developed nausea, vomiting, and right upper quadrant pain in June. He presented to an outside hospital, was diagnosed with cholecystitis, and underwent a laparoscopic cholecystectomy in early July 2013. Although he was reportedly eating normally before and after the cholecystectomy, he had unintentionally lost 40 pounds in the last few months and approximately 70 pounds over the past year (possible causes were unknown to the patient and family). He as well as his family denied alcohol consumption or smoking. Family history was noncontributory for cancer or any diseases. The patient denied recent vaccinations or infections. The patient was admitted to the outside hospital in late July 2013 with a diagnosis of gastritis after his cholecystectomy and was discharged on Reglan for nausea and vomiting. At time of discharge, his mother reported that he was unsteady on his feet and was apparently grabbing at objects that were not present. He was readmitted to the outside hospital the following day and had two tonic-clonic seizures (broken by Ativan). Head CT and chest X-ray at the outside hospital were unremarkable. Lumbar puncture there reportedly showed no WBCs/RBCs, glucose 104, and protein 125. Additionally, his laboratory values were significant for transaminitis (AST 41, ALT 207; remainder HFP normal) and leukocytosis (WBC 15.0). Alcohol level was not significant, but HIV-ELISA was also reportedly positive at the outside hospital. He had an MRI at the outside hospital, which was read as a few nonspecific deep white matter changes.

Due to need for acute care monitoring, the patient was transferred to UPMC Mercy Hospital. On admission to the intensive care unit, the patient was protecting his airways and was intubated; pupils were intact and eyes blinked to visual threat, but the patient did not respond to any commands nor was verbalizing. He moved all extremities spontaneously but very little to stimuli. There was no clonus or Babinski. A stat EEG failed to show epileptiform discharges but did show generalized slowing. Keppra 1 g IV BID was started for prevention of the seizures as well as empiric vancomycin/Zosyn and acyclovir for possible infectious meningitis given his leukocytosis. Repeat HIV-ELISA at our hospital was negative, as was UDS. General surgery was consulted regarding possible intraabdominal pathology or other cholecystectomy-related cause of infection. An abdominopelvic CT scan was entirely unremarkable; however, the surgical service deemed no surgical intervention.

**Figure 1:**
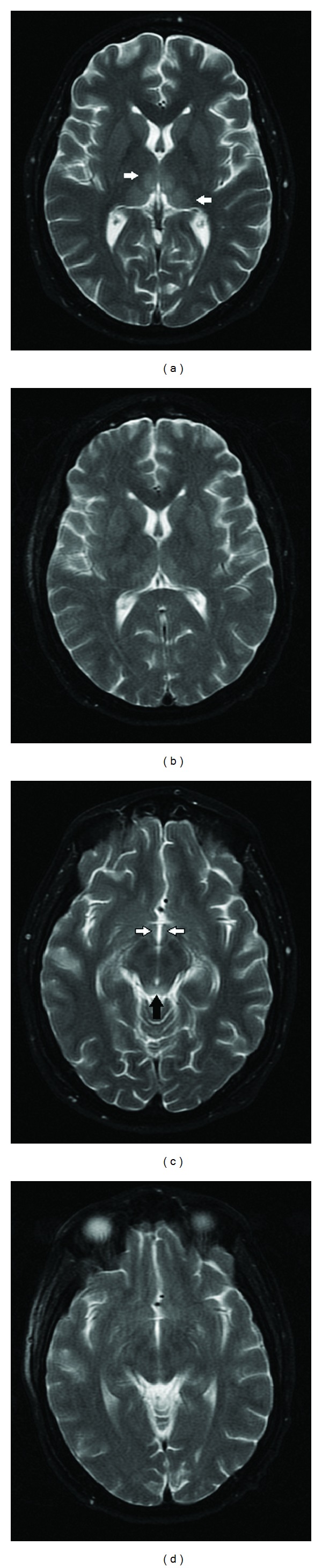
T2 brain MRI one day ((a), (c)) and seven days ((b), (d)) after admission. Signal abnormalities in bilateral thalami ((a), arrows) and interval improvement (b). Signal abnormalities involving the hypothalamus ((c), white arrows), periaqueductal gray matter ((c), black arrows), and interval improvement (d).

**Figure 2:**
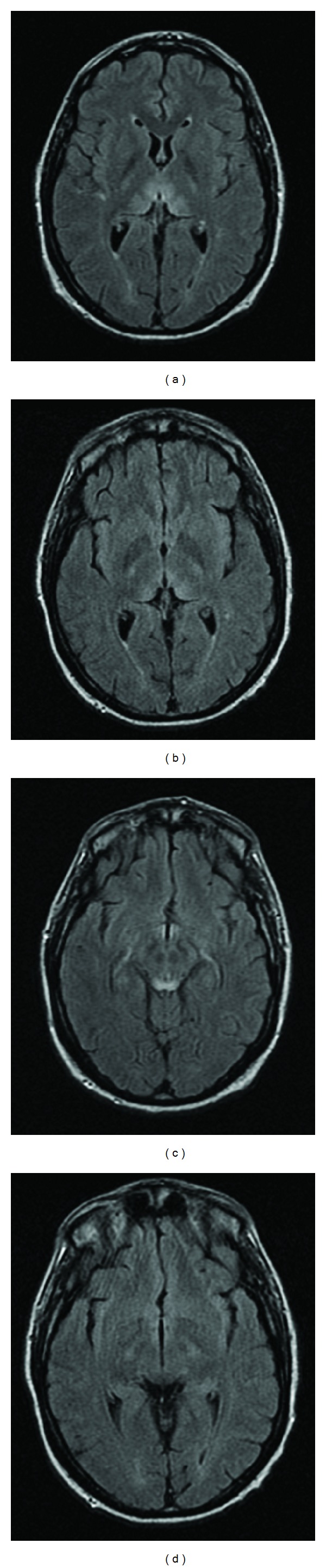
Corresponding FLAIR MRIs one day ((a), (c)) and seven days ((b), (d)) after admission. Signal abnormalities in bilateral thalami (a) and interval improvement (b). Signal abnormalities involving the hypothalamus (c), periaqueductal gray matter (c), and interval improvement (d).

**Figure 3:**
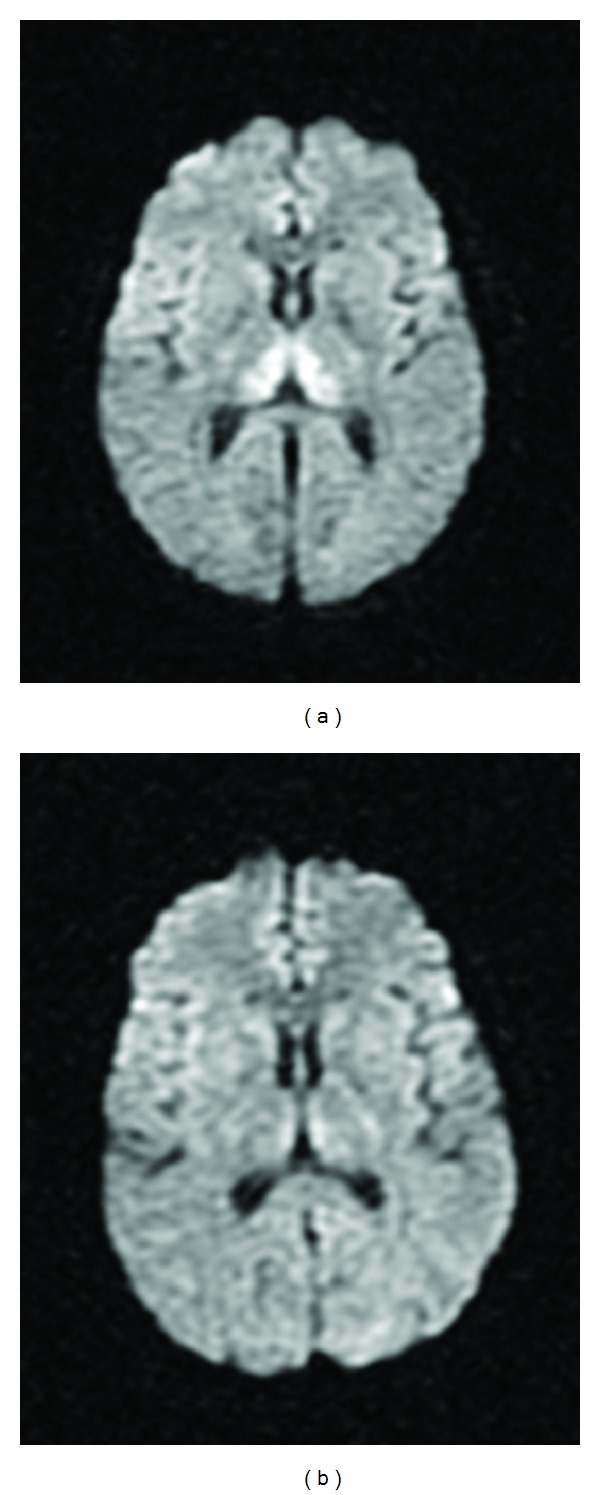
Corresponding DWI MRIs showing bilateral thalamic signal abnormalities one day (a) and seven days (b) after admission.

On admission day 2, the neurological team ordered a follow-up brain MRI, which showed signal abnormalities involving the hypothalamus, periaqueductal gray matter, and bilateral thalami (Figures 1(a), 1(c), 2(a), 2(c), and 3(a)). In consideration of the radiologic findings, the prioritized differential diagnosis included Wernicke's encephalopathy, possibly acute disseminated encephalomyelitis, and less likely CNS vasculitis or less common infections.

Because this list contained Wernicke's encephalopathy, empiric IV thiamine 500 mg TID was immediately started. At this point, after joint discussion with the neurology, infectious diseases, and critical care services, it was agreed that the patient should be started on a trial of 1 g IV methylprednisolone. It was jointly concluded that therapy neither alone nor in combination would foreseeably harm the patient neurologically or immunologically. An NG tube was also placed for general nutrition since the patient had been having weight loss and intractable vomiting.

To entertain the possibility of rare infections, the infectious diseases service conducted further workup. This workup was negative for California, St. Louis, Equine, and West Nile encephalitis. In addition, workup was negative for measles, mumps, LCMV, CMV, Cryptococcus, JC virus, Mycoplasma, RMSF, typhus, and hepatitis viruses. Subsequently, HIV Western blot was negative (CD4 count was normal as well), as were TB tests and ANA.

At this point, levels of vitamin B1 were found to be low at 36 (normal range 78–185); vitamin B12 and folate levels were normal. The patient was more responsive and was responding to commands of opening eyes and squeezing fingers. Now as he could follow commands, when asked to laterally and medially move his gaze, bilateral nystagmus was seen on both lateral and medial gaze deviations. However, because he was responding quite well to current therapy, he was kept on corticosteroids for two more days before a taper started. A repeat lumbar puncture three days after admission showed glucose of 85, decrease in the protein to 97, 3 WBCs, and 103 RBCs. CSF VDRL, HSV-1/2 PCR, and Lyme titers were negative.

Over the next few days, the patient slowly became more responsive to commands and verbal cues and was soon extubated. A repeat MRI on the 7th day of admission showed marked resolution in the abnormal signals (Figures 1(b), 1(d), 2(b), 2(d), and 3(b)). He was transferred to the general medicine floors six days after initial admission. At this point, he was gingerly phonating but was still having problems with salivary secretions; he remained responsive to commands. His mental status gradually improved to the point that he started talking and tolerated a dysphagia diet without nausea. He was then transferred to inpatient rehabilitation 14 days after initial admission. Over the next few weeks, the patient continued to steadily improve to a fully conversational state and essentially normal diet, ambulating well with a walker and with intact cognition.

## 3. Discussion

Wernicke's encephalopathy is a rare finding in nonalcoholic patients; however, the presence of several risk factors such as weight loss and vomiting in this patient was pivotal to focus the differential diagnosis in this patient, along with radiological findings.

Importantly, though thiamine was important for neurological recovery, there is no way of assessing the specific effect of corticosteroids. Unfortunately, there also have been no patient trials in the literature about dual thiamine-corticosteroid therapy versus thiamine alone for Wernicke's encephalopathy, so the additive effect of corticosteroids indeed remains debatable. However, we decided through careful interphysician discussion to proceed with corticosteroids, despite the dearth of evidence related to corticosteroid therapy in Wernicke's encephalopathy, with the goal of decreasing focal cerebral inflammation and edema [4]. Hence, a very salient point from this case is that, after retrospective analysis, we can be relatively certain that this patient tolerated corticosteroids well with no observed harm. Since there is no patient harm with corticosteroids, it is thus our hope that there will be eventual studies (with higher sample sizes) of thiamine with or without corticosteroids for treatment of Wernicke's encephalopathy.

In the present case, therapy produced rapid functional and imaging-based recovery mere days after administration. Though there is no fixed time until MRI signal resolution, the MRI abnormalities generally resolve within a few weeks at minimum [5]. Thus, though it is important to exercise clinical prudence, physicians should not be deterred from therapies that have been poorly described in the literature if these therapies do not carry any significant risk of harm to the patient.

Another aspect of this patient's presentation to discuss is the differential diagnosis of Wernicke's encephalopathy versus ADEM. Though the radiologic findings were vastly more characteristic of Wernicke's encephalopathy [6], ADEM could initially never be fully excluded, though the patient never had any recent infections or vaccinations. History of vomiting and weight loss, as well as cholecystectomy (albeit laparoscopically performed), is associated with Wernicke's encephalopathy; ophthalmoplegia (especially with lateral gaze) and initial presentation of ataxia before his first seizure point more to Wernicke's encephalopathy as well. On the converse side, Wernicke's encephalopathy usually does not resolve as quickly as was seen in this patient. Indeed, ADEM is relatively responsive to corticosteroids and full recovery can be seen as early as a month, both radiologically and functionally [7]. Though, in this patient, thiamine levels were low at 36 ng/mL, in one report, half of patients with Wernicke's encephalopathy had normal thiamine levels [8].

The literature has attempted to correlate brain MRI findings with clinical severity in nonalcoholic Wernicke's encephalopathy [9]. As per this research paper, every patient examined who was comatose had bilateral thalamic damage, and those who were not comatose had damage essentially limited to the periaqueductal gray matter. The patient's clinical presentation stands apart from this trend, since he had bilateral thalamic signal abnormalities but was not comatose (most notably was moving all four limbs spontaneously, albeit little). Therefore, in the case of this patient, the brain MRI severity in a way overstated his clinical symptoms; prediction of a patient's clinical status is thus best done with continuous monitoring and a careful neurologic exam with subsequent radiologic supplements.

The present case serves to show that a nonalcoholic patient presenting with red-flag risk factors of Wernicke's encephalopathy such as persistent prolonged vomiting, unintentional weight loss, and even minimally invasive gastrointestinal surgery should be empirically treated as an atypical case of Wernicke's encephalopathy. Additionally after careful discussion, atypical and out-of-the-box therapy, such as corticosteroids, should not be excluded because it does not cause patient harm. The safe use of corticosteroid therapy in this case, though not evidence based secondary to lack of rigorous study, provides interesting discussion as to whether it should be routinely given for encephalopathic inflammation and cerebral edema.
